# Online Rheometry Investigation of Flow/Slip Behavior of Powder Injection Molding Feedstocks

**DOI:** 10.3390/polym11030432

**Published:** 2019-03-06

**Authors:** Daniel Sanetrnik, Berenika Hausnerova, Vladimir Pata

**Affiliations:** 1 Dept. of Production Engineering, Faculty of Technology, Tomas Bata University in Zlin, nám. T. G. Masaryka 5555, 760 01 Zlín, Czech Republic; dsanetrnik@utb.cz (D.S.); pata@utb.cz (V.P.); 2 Centre of Polymer Systems, University Institute, Tomas Bata University in Zlin, Trida T. Bati 5678, 760 01 Zlín, Czech Republic

**Keywords:** powder injection molding, feedstock, online rheometer, wall slip, slit die, surface roughness

## Abstract

Wall slip in the flow of powder injection molding (PIM) compounds can be the cause of unrealistically low viscosity values, and can lead to a failure of flow simulation approaches. Regardless of its importance, it has been considered only scarcely in the rheological models applied to PIM materials. In this paper, an online extrusion rheometer equipped with rectangular slit dies was used to evaluate the slip velocity of commercial as well as in-house-prepared PIM feedstocks based on metallic and ceramic powders at close-to-processing conditions. The tested slit dies varied in their dimensions and surface roughness. The wall-slip effect was quantified using the Mooney analysis of slip velocities. The smaller gap height (1 mm) supported the wall-slip effect. It was shown that both the binder composition and the powder characteristic affect slip velocity. Slip velocity can be reduced by tailoring a powder particle size distribution towards smaller particle fractions. The thickness of the polymer layer formed at the channel wall is higher for water-soluble feedstocks, while in the case of the catalytic polyacetal feedstocks the effect of surface roughness was manifested through lower viscosity at smooth surfaces.

## 1. Introduction

Powder injection molding (PIM) currently gains enhanced attention due to its merging with additive manufacturing. The PIM process consists of four steps whose accomplishment allows for the production of small and complex-shaped metal or ceramic parts that is often hardly achievable with traditional metallurgical methods [1,2]. During the first step, homogeneous highly filled polymer melt (feedstock) is prepared by compounding metal/ceramic powder into a polymer binder. The binder is an at least three-component system ensuring processability of feedstocks by injection molding, which is followed by its removal from injected parts. This step is called debinding, and can be thermal, solvent, or thermal/solvent combined. Finally, a porous powder structure is sintered to its final density.

Highly filled polymers such as those used in PIM technology belong to rheologically complex systems. Although PIM compounds are high-viscosity (typically 10^3^–10^5^ Pa∙s [1]) materials, many published studies report their viscosity values in the order of flowability of low-molecular weight fluids (tens and hundreds Pa∙s) [3,4]. Such low viscosity values can be attributed to a wall slip appearing during the flow in capillaries. Rheological characterization is essential for the process optimization; however, a no-slip condition is set in most rheological models and simulations of the injection molding step of PIM [5]. 

According to Denn [6], all highly filled materials slip at the wall at certain rates and stresses. Delime and Moan [7] showed that during shearing, a low-viscosity polymer layer with a typical thickness of 0.1–10 µm is formed near flow channel walls as powder particles migrate away from walls during flow [6,8,9]. Kalyon and Aktas [10], as well as Soltani and Yilmazer [11], reported that the thickness of the layer is about 1/14–1/25 of a capillary diameter. This is known as an apparent slip [12]. Delime and Moan, in the abovementioned study [7], further suppose that the migration of solid particles is initiated by the failure of Brownian movement near the walls, which is supported by shear rate gradients which promote particle collision. For PIM feedstocks, Thornagel [13] assumes that particles present in the regions of high shear rate gradients try to leave these areas and concentrate in the middle of the flow channel, which results in so-called powder/binder separation. [14,15]. 

Soltani and Yilmazer [11] tested the tendency of hydroxyl-terminated polybutadiene (HTPB) filled with glass spheres to wall slip on a parallel-plate rheometer, and found the slip layer thickness to be independent of temperature, but enhanced with the size of the particles. On the other hand, the slip velocity increased with increasing temperature, which was attributed to lower polymer viscosity. Increasing wall-slip velocity with rising particle size was reported by Gulmus and Yilmazer [16] for poly(methyl methacrylate) (PMMA) particles in HTPB as a result of the steric hindrance effect of the particles—larger particles cannot get very close to the wall and this causes the slip layer thickness (and consequently slip velocity) to increase. 

Concerning the effect of the processing tool (the chemical nature of materials and roughness), Chen et al. [17] tested linear low-density polyethylene (LLDPE) and concluded that its wall-slip velocity increases in the following manner: aluminum < glass < copper < stainless steel. As can be seen, the stainless-steel die, which is mostly used for processing tools, is prone to wall slip. This was attributed to a relatively small work of adhesion, and also a smooth capillary surface in comparison to a rough surface of aluminum, which improved the material’s adhesion to the wall. A similar effect of surface roughness was confirmed by carrying out studies using poly(butadiene-acrylonitrile-acrylic acid) terpolymer mixes with glass spheres (85.4 and 35.3 μm) [18] or HTPB filled with PMMA [19]. According to Medhi et al. [20], in the case of a rough wall, solid particles can move into the groove, therefore, the whole material flow as a continuum and formation of low molecular layer is suppressed. 

Concerning the geometry of measuring tools, Kalyon [21] proposed methodology to provide the slip-corrected shear viscosity of concentrated suspensions in Couette, capillary, and rectangular slit flows on the basis of the apparent slip mechanism. The influence of the entrance angle of capillary dies was tested recently [22] with the conclusion that the application of conical dies seems to be more beneficial as the corresponding rheological measurements are in accordance with general wall-slip findings. Finally, in the study published by Walter et al. [23], the relation between slip and static axial preload, as well as the measuring frequency of a parallel-plate rotational rheometer, was tested on two-component silicone rubber. The wall-slip velocity was rather sensitive to the applied preload—higher preload force resulted in a delay in the onset of wall slip. On the other hand, only a slight dependence of wall-slip on frequency was found. 

As pointed out by Kwon and Ahn [24], disregarding slip leads to inaccurate simulations of the PIM process. Very recently, the slip of PIM compounds was considered by Liu et al. [25] for micro-PIM of zirconia feedstock with the clear conclusion that it cannot be ignored in numerical simulations. In the following work, Liu et al. [26] supported this finding when comparing the simulations of temperature, viscosity, and pressure gradient distributions during mold filling, including/excluding wall slip.

The present study was performed on an online rheometer with slit dies of different sizes and surface roughness. The purpose of this study is to point out the importance of the wall-slip effect and to show that it is the typical effect for PIM compounds, occurring for the most-often employed PIM feedstocks and, thus, it should always be examined when performing rheological characterization of these highly filled polymers. Concerning PIM compounds, there are—to our best knowledge—no studies considering the dimensions and surface roughness of the processing dies. Also, there are no wall-slip studies performed on commercially available feedstocks. 

## 2. Materials and Methods

### 2.1. Materials

**Figure 1:**
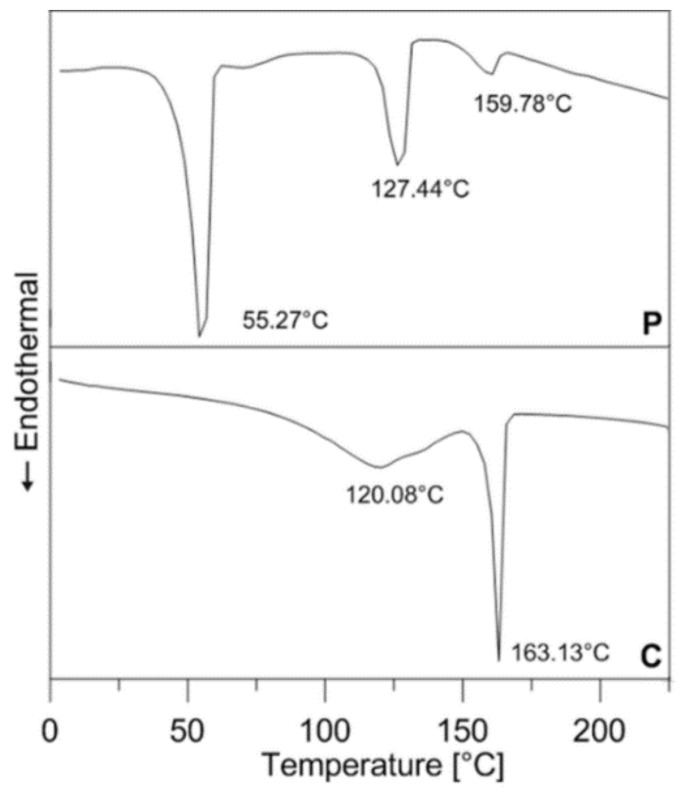
DSC of partly water soluble (P) PolyMIM binder, and catalytic polyacetal (C) Catamold (BASF) binder systems (10 °C/min, second heating scan).

The research covered both in-house-prepared and commercially available PIM feedstocks; their composition can be seen in Table 1. Commercial compounds P316L, P17-4PH, C316L, and C17-4PH were based on gas-atomized stainless steels 316L and 17-4PH, and differed in polymer binders (abbreviated P and C for partly water soluble and catalytic polyacetal binder systems, respectively), as can be seen from the transition temperatures obtained from the second heating scan (Mettler Toledo DSC1 Star, temperature range from 0 to 250 °C, nitrogen atmosphere, 10 °C/min) depicted in Figure 1. All these compounds had powder volumetric concentrations higher than 60%.

On the other hand, ceramic powders—zirconium oxide (ZrO_2_) and aluminum oxide (Al_2_O_3_)—were admixed to an in-house-prepared binder system at the loading level of 50 vol %. Particle size distributions were measured with the laser diffraction particle size analyzer Malvern Mastersizer 3000 (Malvern, UK). The particle size distribution of ceramic powders was (in μm) *D*_10_—0.3, *D*_50_—0.56, *D*_90_—2.5; and *D*_10_—0.19, *D*_50_—0.3, *D*_90_—0.47 for Al_2_O_3_ and ZrO_2_ powders, respectively. Feedstocks produced by PolyMIM exhibited a particle size distribution of *D*_10_—3, *D*_50_—9, *D*_90_—26, and Catamold feedstocks of *D*_10_—7, *D*_50_—15, *D*_90_—28.

### 2.2. Preparation of In-House Feedstocks

First, the critical solid loading of powders was determined with a torque rheometer at 160 °C. In the process, the concentration of powder in the mixing chamber was gradually increased by 1 vol % until an unstable and rapidly increased torque signalized critical solid loading. Then, the feedstocks were mixed on a Brabender plastometer with a twin-screws extruder setup. Along the screw, the temperature changed. In the first zone it was set to 140 °C, in the second zone to 150 °C, and at the end of the screw the temperature was increased to 160 °C. A stable temperature profile during the mixing process was maintained, regardless of high dissipation energy, in order to ensure the homogeneity of the mixtures. Extruded feedstocks were subsequently granulated on a grinding machine. 

### 2.3. Rheological Investigation

For slip evaluation, an online Brabender Extrusion Lab (19/25D) rheometer (Duisburg, Germany) and four slit dies of different dimensions and surface roughness were employed, as demonstrated in Table 2. For ceramic powder feedstocks, which are generally considered to be more abrasive than metal-based compounds, we used higher surface roughness and modified geometry of slit dies. The surface roughness of the dies was quantified with an optical surface profiler ZYGO NEWVIEW 9000 (Berwyn, PA, USA) based on coherence scanning interferometry, which provides non-contact real topography maps of smooth, highly reflective surfaces of sub-10 nm roughness. In Table 2, the channel surface properties are described with the 3D roughness parameter *S*a.

**Figure 2:**
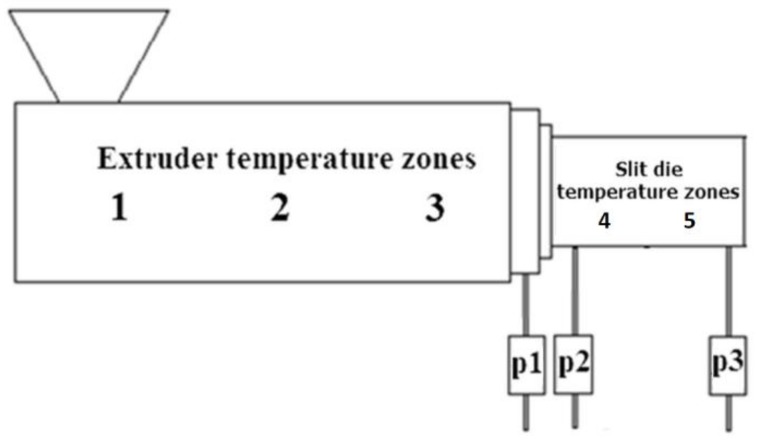
Scheme of the testing online rheometer.

The measurements were carried out on an online rheometer, schematically presented in Figure 2. 

As can be seen, the pressure profile was detected by three pressure transducers. For metal powder feedstocks, the individual screw zones (1 to 3) were set to 170, 180, and 190 °C, respectively, and in the slit die (zones 4 and 5) the temperature was 190 °C. For ceramic powder feedstocks the corresponding temperature profile was (140–150–160–160–160) °C. 

The method for wall-slip evaluation from capillary data was proposed by Mooney as early as in 1931 [27]. The slip velocity was calculated from the slope of the apparent shear rate as a function of the reciprocal capillary radius data determined at the constant apparent wall shear stress. In order to obtain experimental data at close-to-processing conditions, this method was modified by Kalyon et al. [28] for measurements on an online slit die rheometer with an adjustable die gap. 

In calculations for on an online rheometer, the shear stress *τ* (Pa) was derived from

(1) $$\tau =\frac{\left(p_{2}-p_{3}\right)H}{2L}$$

where *H* (mm) represents the height of the slit die and *L* (mm) is the length between the transducers *p*_2_ and *p*_3_ (Figure 2). 

The shear rate was calculated as

(2) $${\dot{\gamma }}_{a}=\frac{6\dot{Q}}{WH^{2}}$$

where W (mm) is the width of the slit die, and $\dot{Q}$ (mm^3^/s) stands for the volumetric flow rate. 

**Figure 3:**
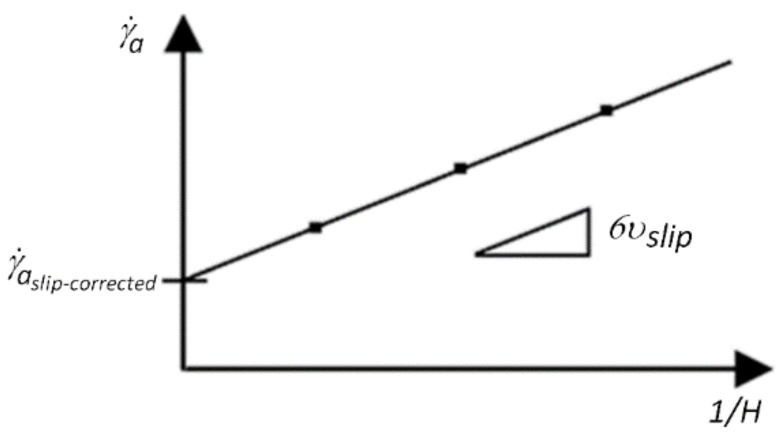
Calculation of wall-slip velocity.

Figure 3 demonstrates the calculation of the wall-slip velocity according to the Mooney method. 

The volumetric flow rate $\dot{Q}$ is related to the calculated average velocity $\upsilon_{av}$ in the capillary die as

(3) $$\nu_{av}=\frac{\dot{Q}}{WH}$$

The calculated average velocity $\upsilon_{av}$ in the die is related to the slip-corrected average velocity $\upsilon_{true}$ through the slip velocity $\upsilon_{slip}$ as

(4) $$\upsilon_{true}=\upsilon_{av}-\upsilon_{slip}$$

To obtain the relationship between the slip-corrected apparent shear rate ${\dot{\gamma }}_{a,\;slip-corrected}$ and the measured apparent shear rate ${\dot{\gamma }}_{a},$ we have to multiply by 6/H:

(5) $${\dot{\gamma }}_{a}=\frac{6\upsilon_{av}}{H}=\frac{6\dot{Q}}{WH^{2}}$$

(6) $$\frac{6\upsilon_{true}}{H}=\frac{6\upsilon_{av}}{H}-\frac{6\upsilon_{slip}}{H}$$

Then, after rearranging, we obtain

(7) $${\dot{\gamma }}_{a,slip-corrected}=\frac{6\dot{Q}}{WH^{2}}-\frac{6\upsilon_{slip}}{H}$$

## 3. Results and Discussion

**Figure 4:**
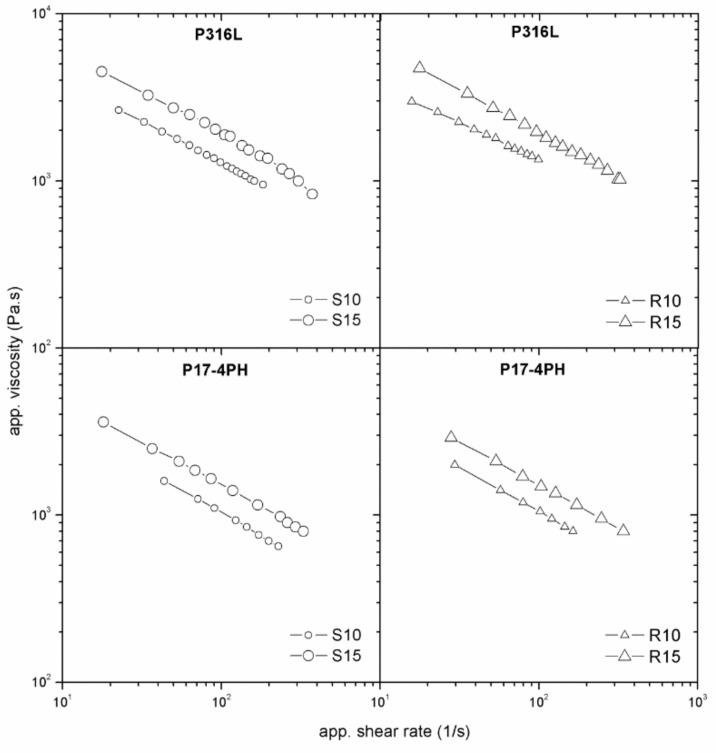
Effect of die dimensions on flow properties of stainless-steel powders 316L (P316L) and 17-4PH (P17-4PH) in the partly water-soluble binder system PolyMIM (P).

The dependence of viscosity on the surface roughness and die geometry is an indicator of wall slip occurring in the flow of highly filled compounds. All tested PIM feedstocks exhibited slip during shear deformation. The feedstocks based on stainless-steel powders (Figure 4) showed the same trend in the slip dependence on die dimension regardless of surface roughness. However, the feedstock with the partly water-soluble binder (P316L and P17-4PH) shows a stronger influence of flow channel geometry on viscosities than that with the catalytic binder, indicating an enhanced tendency to slip. 

**Figure 5:**
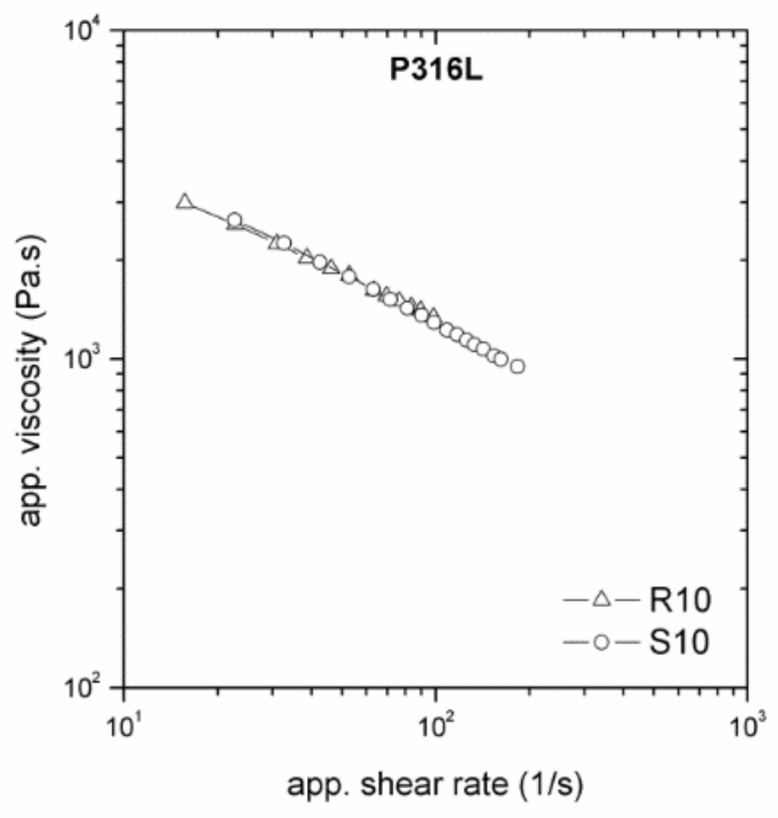
Effect of surface roughness on flow properties of P316L, stainless-steel powder 316L, in the partly water-soluble binder system PolyMIM (P).

Let us move now to the effect of surface roughness. As can be seen from Figure 4 (R10, R15) and Figure 5, the presence of a water-soluble binder in the feedstock resulted in an imperceptible influence of the surface roughness. This means that the thickness of the polymer layer formed at the channel wall was higher than the surface irregularities [12,20] of the tested slit dies. 

**Figure 6:**
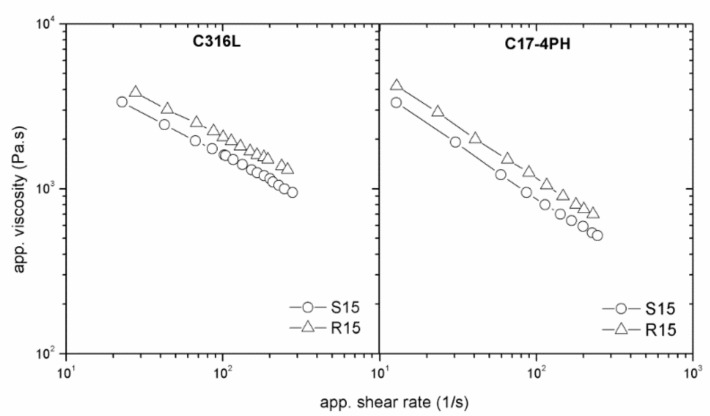
Effect of surface roughness on flow properties of stainless-steel powders 316L (C316L) and 17-4PH (C17-4PH) in catalytic polyacetal Catamold binder system (C).

On the other hand, for both types of metal powders in the catalytic binder (Figure 6), the effect of surface roughness was evident—a lower viscosity was obtained for a smooth surface. The observed trend is in agreement with the simulations performed by Papanikolaou et al. [29], where parallel liquid layers that formed near a smooth wall surface were disturbed in case of a roughened surface (leading to a higher viscosity). 

Furthermore, we compared the effect of the rheometer type. For metal powder feedstocks, the slip behavior obtained on an online rheometer corresponded with our previous findings [22] achieved on a capillary rheometer. Feedstocks based on a partly water-soluble binder exhibited a higher wall-slip velocity (a thicker polymer layer formed on the flow channel wall) in comparison with catalytic feedstocks, where a lower wall-slip velocity was observed. In this case, however, due to the considerable pressure fluctuations, (10 × 0.5 × 100) mm geometry has been disregarded for catalytic feedstocks, where, similarly to ceramic feedstocks, for further testing the gap was enlarged to (10 × 1 × 100) mm. 

**Figure 7:**
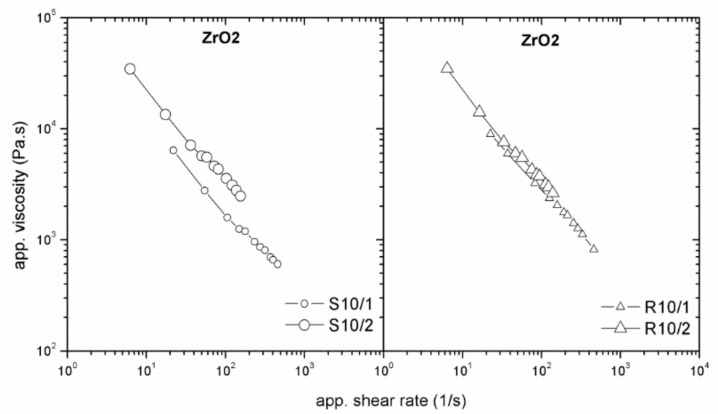
Effect of surface roughness on the flow properties of zirconium oxide (ZrO_2_)/LDPE+EVA+PW feedstock.

Using roughened surfaces of the processing tools should diminish, or even eliminate, wall slip because powder particles (in case of an apparent slip) or polymer chains (in case of true slip) can move into the grooves, polymer layer is eliminated, and the suspension can flow as a continuum [20]. For pure polymer melts, this theoretical finding was confirmed for LLDPE, where a smooth capillary surface revealed the wall-slip velocities 50 % to 150 % greater than roughened dies [17]. In case of highly filled compounds, we obtained the same trend in testing ZrO_2_ feedstocks (Figure 7) where, due to higher abrasion of ceramics, the surface roughness was increased for the smooth as well as for the rough surface, to capture the difference. 

**Figure 8:**
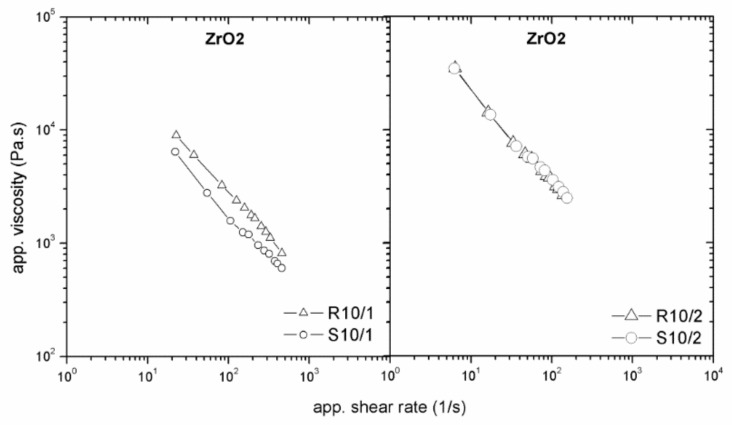
Effect of die dimension on the flow properties of zirconium oxide (ZrO_2_)/LDPE+EVA+PW feedstock.

Another factor influencing wall slip could be the die geometry. As can be seen from Figure 8, the smaller gap height (1 mm) supported the wall-slip effect, while this was substantially reduced for a 2 mm gap. 

These measurements again correspond to findings on pure linear low-density polyethylene in the capillary flow with different surface roughness [17]. Further, this behavior is in agreement with that of suspensions containing polymer matrix poly(butadiene-acrylonitrile-acrylic acid) filled with glass spheres (particles with a mean size of 35.3 m and 85.4 m) [18] and poly(methyl methacrylate) solid spheres (121.2 m) in hydroxyl-terminated polybutadiene [16], where smooth flow channel walls and small channel geometry [8,30] were found to be the most significant factors causing wall slip. 

**Figure 9:**
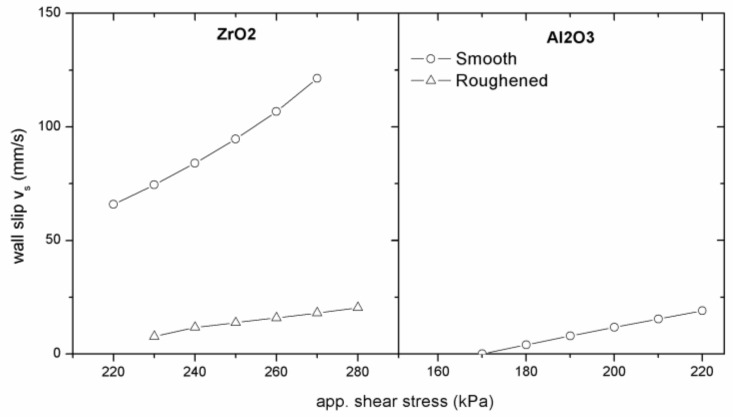
The effect of surface roughness on wall-slip velocity of zirconium oxide (ZrO_2_) and aluminum oxide (Al_2_O_3_) powders in LDPE+EVA+PW binder.

The influence of the surface roughness on the slip velocity for ceramic powder feedstocks is presented in Figure 9. 

As can be seen, the slip velocity in the roughened die is substantially lower than in the smooth-wall die. The measurement of the rheological behavior in smooth dies results in greater underestimated viscosity, however, as shown in the studies [13,14], due to lower shear rate gradients, slipping at the wall might positively affect the tendency of PIM feedstocks towards powder and binder separation. Figure 9 demonstrates that the values of slip velocities obtained for Al_2_O_3_ powder in the smooth slit die are comparable with the slip velocities of ZrO_2_ feedstock in the roughened slit die; the tendency of Al_2_O_3_ feedstock to wall slip as a function of the die geometry and surface roughness is generally less pronounced than for ZrO_2_ compound, although as we showed recently [31], their surface characteristics (surface energies) were fairly similar—44 and 47 J/m^2^ for ZrO_2_ and Al_2_O_3_, respectively. 

## 4. Conclusions

Online rheological investigation of PIM compounds was focused on the wall-slip phenomenon. As could be seen, all tested highly filled materials exhibited wall slip. Thus, neglecting this phenomenon may lead to inaccurate flow data and, as a consequence, to non-realistic simulations of the injection molding step of the PIM process. The results reveal the importance of surface roughness to wall-slip development if surface irregularities are higher than polymer layers formed at flow channel walls. Generally, the wall-slip velocity decreases with increasing surface roughness. Furthermore, the higher tendency of materials to wall slip was observed in smaller dies. Therefore, both parameters—surface roughness and geometry of the flow channels—are important for reliable testing of highly filled materials. 

## Figures and Tables

**Table 1:** Investigated powder injection molding (PIM) feedstocks.

Abbreviation of Feedstock	Type of Powder	Type of Binder	Commercial Name	Producer
P316L	Stainless steel 316L	Partly water soluble (PEG based)	PolyMIM 316L D 110 E	PolyMIM GmbH
P17-4PH	Stainless steel 17-4PH	Partly water soluble (PEG based)	PolyMIM 17-4PH D 110 E	PolyMIM GmbH
C316L	Stainless steel 316L	Catalytic (Poly-acetal based)	Catamold 316 L G	BASF
C17-4PH	Stainless steel 17-4PH	Catalytic (Poly-acetal based)	Catamold 17-4PH G	BASF
ZrO_2_	Ceramic ZrO_2_	LDPE ^1^ + EVA ^2^ + PW ^3^	in-house	see bellow
Al_2_O_3_	Ceramic Al_2_O_3_	LDPE ^1^ + EVA ^2^ + PW ^3^	in-house	see bellow

^1^ ExxonMobil™ LDPE LD 650 Low Density Polyethylene Resin, ^2^ ExxonMobil™ Escorene™ Ultra UL 40028CC Ethylene Vinyl Acetate Copolymer Resin, ^3^ FAGRON Paraffinum solidum

**Table 2:** Characteristics of slit dies used.

Metal Powder Feedstocks			Ceramic Powder Feedstocks		
Geometry (mm)	Surface	Roughness *S*a (µm)	Geometry (mm)	Surface	Roughness *S*a (µm)
10 × 0.5 × 100	smooth	0.25 ± 0.03	10 × 1 × 100	smooth	0.81 ± 0.03
	roughened	0.95 ± 0.02		roughened	9.65 ± 0.18
15 × 1 × 100	smooth	0.07 ± 0.00	10 × 2 × 100	smooth	0.82 ± 0.04
	roughened	0.77 ± 0.03		roughened	7.87 ± 0.70
